# Exploring the Relationship between Academic Challenge Stress and Self-Rated Creativity of Graduate Students: Mediating Effects and Heterogeneity Analysis of Academic Self-Efficacy and Resilience

**DOI:** 10.3390/jintelligence11090176

**Published:** 2023-09-04

**Authors:** Hao Yao, Shuzhen Chen, Ang Liu

**Affiliations:** 1 Institute of Higher Education, Tongji University, Shanghai 200092, China; yaohao@tongji.edu.cn; 2 Faculty of Education, East China Normal University, Shanghai 200062, China; liuang@stu.ecnu.edu.cn

**Keywords:** challenge academic stress, self-rated creativity, resilience, academic self-efficacy

## Abstract

In the 21st century, creativity is a core competence and key thinking quality. Researchers and educators have been interested in exploring the effects of different stressors on individual creativity for decades. Using structural equation modeling and quantile regression, this study investigated the relationship between academic challenge stress and self-rated creativity of graduate students among 1210 Chinese graduate students. The study separately tested the mediating effect of resilience, the mediating effect of academic self-efficacy, and the chained mediating effect of both. This study analyzed the heterogeneity of the effects of academic challenge stress, academic self-efficacy, and resilience on self-rated creativity of different students. The research results showed that academic challenge stress had a direct positive effect on graduate students’ self-rated creativity. The mediating effect of resilience and academic self-efficacy and the chain mediating effect were established. The quantile regression revealed a decreasing marginal benefit of academic challenge stress and resilience for self-rated creativity and an inverted U-shaped relationship between academic self-efficacy and self-rated creativity.

## 1. Introduction

Creativity, a multidimensional construct, is commonly defined as the production of novel and useful ideas or solutions (Amabile 1983). The quality of graduate student cultivation, especially their level of creativity, largely determines the quantity and quality of innovative talents. Therefore, improvement of graduate student creativity is also considered as an important indicator for evaluating the quality of higher education (Chan and Ngok 2011). How to cultivate and stimulate graduate student creativity and thus enhance the innovation capacity and performance of universities has gradually become the focus of universities (Shu et al. 2022). However, creativity as an important feature of graduate student performance has received little academic attention (Frick and Brodin 2020).

Previous studies have mostly analyzed the factors influencing creativity in terms of psychological and organizational factors (Anderson et al. 2014; Standing et al. 2016). But research stressing creativity is not very mature, and scholars often take stress as a negative factor affecting individual abilities. Indeed, there has been no consistent conclusion on the theoretical attributions, effects, and underlying mechanisms of different stressors affecting creativity (Byron et al. 2010; Cai et al. 2022). Hon and Lui (2016) suggest that stress deserves more attention as a key factor in creativity. Current research on the effects of stress on individual creativity can be divided into positive functional theory, negative stress theory, and stress balance theory. Functionalism is based on the idea that “stress is motivation”. That is, stress can motivate individuals’ sense of achievement and efficacy. This is because they see difficult tasks as a great opportunity to improve their skills and knowledge. Moreover, when they are under great stress, they are stimulated to be more challenged, and individuals have higher levels of positive motivation in trying to achieve their goals (LePine et al. 2004). In other words, the process of overcoming stress can awaken creativity (Bunce and West 1994; Yeh et al. 2015). Negative stress theory is a pessimistic perception that stress depletes cognitive resources. Individuals facing external pressures may experience work overload, role ambiguity, and role conflict and perceive that they do not have abilities to perform satisfactorily at work, which may result in stress reactions that cause individuals to experience physical and psychological symptoms (Devereux et al. 2009) and even weaken creativity (Talbot et al. 1992; Grosser et al. 2018). The equilibrium theory suggests an inverted U-curve relationship between stress and creativity, with the right level of stress maximizing creativity (Baer and Oldham 2006; Shao et al. 2019; Antwi et al. 2019). In response to the findings that stress affects creativity differently, a specific categorization framework of challenge versus blocking stress has been proposed, i.e., there are also “good” and “bad” categories of stress, and differences in the nature of stress may be a key factor in explaining differences in creativity outcomes (Byron et al. 2010; Byron et al. 2018). Challenge stress may originate from positive stressors. Challenge stress comes from the environment, such as competition, work, study, family, etc. It can also come from internal motivation, such as self-motivation, self-challenge, self-actualization, etc. Challenge stressors can help people attain higher achievement, stimulate their potential, and promote positive psychological experiences after individuals achieve their goals (LePine et al. 2005, 2016).

Little research on the relationship between challenge stress and creativity has focused on the field of graduate education. Zhao et al. (2021) analyzed the effect of advisor–student relationships on student creativity, analyzing challenge stress as a mediating variable. Eisenberg and Thompson (2011) analyzed the role of stress of students as a mechanism influencing creative performance during improvisation. However, there is a lack of research on academic challenge stress and the creativity of graduate students. Academic challenge stress focuses on the academic field and refers to positive stress in terms of academic responsibilities, academic tasks, academic goals, and academic workload.

The relationship between challenge stress and creativity may be influenced through the mediating role of resilience (Liang et al. 2021) and academic self-efficacy (Zhang et al. 2018). Resilience refers to a person’s ability to coordinate and adapt psychologically when they are facing adversity such as difficulties, setbacks, and failures (Holdsworth et al. 2018). It relates to an individual’s ability to recover from risk and stress and has been widely recognized as an important competency for graduate students’ development (Van Kessel et al. 2022). Self-efficacy refers to an individual’s judgment of his or her ability to organize and perform to achieve desired goals (Bandura 1997). Academic self-efficacy is self-efficacy in a particular academic field. Academic self-efficacy is defined as the learner’s judgment of one’s ability to successfully achieve academic outcomes in a given academic context (Elias and MacDonald 2007). It is more specifically regarded as a graduate student’s perceived ability to successfully deal with different curriculum, learning activities, academic research projects, and faculty–student peer relationships (Greco et al. 2022).

This study of the relationship between academic challenge stress and creativity is unique to Chinese graduate students. According to statistics from the Chinese Ministry of Education, there were 3.33 million graduate students in China in 2021. Unfortunately, with such a large graduate student population, Chinese graduate students lack creativity education, and creativity research has not received sufficient attention (Pang and Plucker 2012). In addition, Chinese graduate students face complex academic stress. Intense academic competition has transferred the stress to graduate students, which has significantly increased the stress on graduate students in terms of academic performance and publication (Li 2016; Wang et al. 2019). Actually, current research on graduate students’ academic stress in the Chinese context does not strictly distinguish between challenge stressors that have positive effects and hindering stressors that have negative effects.

The goal of our study is to empirically explore the relationship between academic challenge stress and self-rated creativity among Chinese graduate students and to verify the mediating effect of resilience and academic self-efficacy. Meanwhile, we want to examine the variability of the effects of academic challenge stress, resilience, and academic self-efficacy on graduate students with different levels of self-rated creativity through quantile regression models. This study contributes to helping scholars understand the relationship between academic challenge stress and self-rated creativity and suggests that different stress strategies need to be provided to different graduate students in order to enhance their creativity.

Existing studies have focused on the effects of challenge stress on employee creativity in the field of business management (Geng et al. 2014; Sun et al. 2019; Zhang et al. 2019). Our research focuses on graduate students’ challenge academic stress and self-rated creativity aiming to expand the scope of application for previous studies on stress on individual self-rated creativity and enrich the relevant literature.

### 1.1. Academic Challenge Stress and Creativity

Most graduate students face two differential outcomes when they suffer from academic stress. The first one is that academic stress drives graduate students to engage in academic activities and prompts them to enhance their creativity (Sun et al. 2019). Another outcome is that the academic stress seriously increases the psychological burden of graduate students, leading to a negative psychological state and resistance, which may also breed academic corruption and academic misconduct, not to mention the inability to enhance individual creativity (He and Wong 2015). The reason for the two different results on the impact on graduate student creativity is that there are differences in the types of stressors. LePine et al. (2004) explain that stressors can be categorized into challenging and hindering stressors based on differences in their attributes. Although stress sounds slightly negative, academic challenge stress appears to be a positive element because it is understood to be stress that individuals believe they can overcome and that is beneficial to their own work performance and academic development. Taking on the appropriate stress is accompanied by a corresponding reward and a sense of recognition of the value of the current academic work (Travis et al. 2020). Moreover, academic challenge stress usually has benefits on individual outputs. For example, Zhu et al. (2017) showed that academic challenge stress was positively associated with students’ academic performance. For the purpose of this paper, an example of academic challenge stress in the graduate student context is “I can conduct independent research and complete difficult academic tasks and workloads, and take on challenging academic responsibilities”. 

Previous studies showed that challenge stress has a positive impact on an individual’s life, work, and personal growth (Liu and Ren 2022; LePine et al. 2005). In addition, challenge stress is a positive stressor that enhances innovation performance (Cai et al. 2022; Moin et al. 2022). In terms of the reason why challenge stress affects creativity performance, from a psychological perspective, the Conservation of Resources (COR) Model indicates (Halbesleben et al. 2014) that individuals strive to protect their resources and obtain additional resources in all situations. Challenge stress may largely result in a successful outcome. If a person enjoys challenging and creative tasks, this leads to a spiral of individual resource acquisition, which increases positive emotions and creative performance (Akinola et al. 2019). Kim and Beehr (2018) found that challenge stressors can promote individuals to produce better work behaviors and enhance achievement motivation and psychological empowerment as a way to promote creativity. Akinola et al. (2019) argued that explanations of physiological responses to stress can inspire scholars to understand the relationship between stress and creativity. The effect of stress on creative performance critically depends on whether stress-inducing situations engender challenging physiological states (i.e., fluid physiological stress responses). The transactional model of stress predicts that an individual facing a creative task will first appraise whether engaging in the creative task is likely to be beneficial or harmful (Li et al. 2018). For example, if an individual is faced with the need for creativity to solve a problem, he will judge whether it is worth investing time to solve and overcome the problem based on what is at stake for him in the current situation (Akinola et al. 2019). When graduate students are confronted with academic tasks that require creativity, they show higher levels of creativity when they view the experience of the academic task as a challenge rather than a threat. Therefore, this study proposes Hypothesis 1:

**H1:**  *Higher academic challenge stress is associated with higher self-rated creativity of graduate students.*

### 1.2. The Mediating Effect of Resilience

Garmezy et al. (1984) identified three primary models of resilience including the compensatory model, protective factor model, and challenge model. The challenge model refers to the fact that challenge stress actually increases resilience. Wister et al. (2016) describe resilience as the dynamic process by which individuals use personal, life, and environmental resources to effectively negotiate, adapt, and manage stressors in order to adapt to adversity. Moderate challenge stress, especially when individuals are able to adapt to and overcome such stress, actually contributes more to individual resilience (Smith and van der Meer 1994; McLaughlin et al. 2008).

Thomson (2020) highlights the close relationship between resilience and creativity. Resilient and creative individuals share characteristics such as flexibility, initiative, resourcefulness, adaptability, spontaneity, and originality (McFadden and Basting 2010). It also includes emotional positivity, as individuals with high resilience can cope with critical situations with humor, creative exploration, and optimistic thinking (Fredrickson et al. 2003) and generate higher levels of creativity (López-Aymes et al. 2020). Individuals with high resilience also have higher individual adaptive cognition and divergent thinking patterns (Cranney and Morris 2011), which is a component of creative potential (Runco 2008). Adaptive cognition and divergent thinking patterns enable them to understand problems in complex situations from different perspectives and find possible solutions to overcome various difficulties with creativity (Kashdan and Rottenberg 2010). When graduate students face academic challenge stress, for example, when they choose a research topic, summarize existing empirical evidence in the literature review, and outline a research design, they may undertake the stress of an academic research task with high goals. The stressful process of academic research can help them to develop a certain level of initiative and adaptability to promote resilience. After overcoming this academic stress, they can generate higher achievement, motivation, etc., which promotes the creativity of graduate students (Eisenberg and Thompson 2011). Hence, the study proposes Hypothesis 2:

**H2:**  *Resilience plays a mediating role in the relationship between academic challenge stress and the self-rated creativity of graduate students.*

### 1.3. Mediating Effects of Academic Self-Efficacy

When graduate students face academic challenge stress, the supervisor or organization assigns more academic research tasks to them, which indicates the expectation, trust, and empowerment of the organization and supervisor, and the graduate students are convinced that they are fully capable of achieving their academic research tasks or goals by putting in extra efforts and gaining high moral benefits after achieving their goals, and this social persuasion effect helps stimulate positive psychological states such as the self-efficacy of graduate students (Widmer et al. 2012; Prem et al. 2017). Hence, academic challenge stress can promote academic self-efficacy. 

Social cognitive theory suggests that self-efficacy is an important motivator for individuals to sustain their efforts in the face of challenges to achieve their goals (Gong et al. 2009). Graduate students with high academic self-efficacy are more likely to set challenge goals that change the status quo, generate novel and useful ideas, work hard to achieve their goals, and persevere in the face of difficulties and failures (Liao et al. 2010). Graduate students with high self-efficacy also tend to have the confidence and ability to make a difference and generate creative ideas (Tierney and Farmer 2011). Empirical research also confirms that self-efficacy is a positive predictor of creativity (Liao et al. 2010; Kim et al. 2019). So we propose hypothesis H3:

**H3:**  *Academic self-efficacy plays a mediating role in the relationship between academic challenge stress and self-rated creativity of graduate students.*

### 1.4. The Chain Mediating Effect of Resilience and Academic Self-Efficacy

Research on the relationship between resilience and self-efficacy has been recognized as an important topic (Schwarzer and Warner 2013; Sagone and De Caroli 2016a). According to Hurtes and Allen’s (2001) approach and the revised model of resilience by De Caroli and Sagone (2014), individuals with high resilience have distinguishing characteristics, such as the ability to know how to deal with problems in unfavorable environments or under significant stress. It reflects the tendency of individuals with high resilience to look for the “positive side” of difficult situations. They can manage stress with positive emotion and regulate behavior (Sagone et al. 2020). These characteristics are positively associated with Bandura’s (2007) sense of self-efficacy. As in Hamill’s (2003) study, analyses based on the distribution of students’ levels of resilience indicated that differences in resilience were an important feature in distinguishing students’ self-efficacy. Sagone and De Caroli’s (2016b) study also found that adolescents with high resilience showed greater self-efficacy than those with less resilience in general (and in academic settings in particular). Therefore, this study concluded that graduate student resilience contributes to academic self-efficacy. Meanwhile, resilience underlies an individual’s ability to deal with stress and anxiety (Holdsworth et al. 2018; Brannick et al. 2005), which can be affected by academic stress (Caruana 2014). Academic self-efficacy in turn positively affects individual creativity (Shaabani et al. 2011). So, we propose hypothesis H4:

**H4:**  *Resilience and academic self-efficacy play a chain mediating role between academic challenge stress and the self-rated creativity of graduate students.*

**Figure 1:**
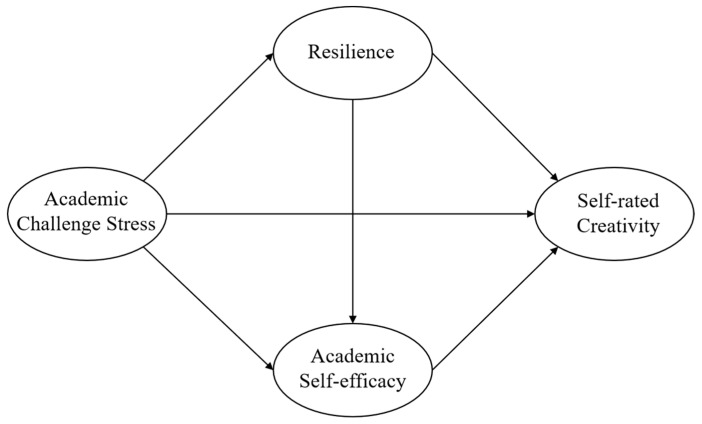
The hypothesized model.

Hence, we construct a chain mediation model to explore the relationship between academic challenge stress and graduate student self-rated creativity and the chain mediation role of resilience (Figure 1).

### 1.5. Heterogeneity of Effects on Creativity

There may be a difference in the effects of stress, resilience, and academic self-efficacy on creativity across individuals. Individuals with high levels of creativity may have different characteristics than those with low levels of creativity, and individuals with higher levels of creativity may have better openness, unconventionality, and ambition (Helson and Srivastava 2002). And these characteristics will cause stress to have differential effects on individuals with different levels of creativity. Therefore, it is necessary to analyze the impact effects on graduate students with different creativity. Furthermore, in terms of the relationship between resilience and creativity, López-Aymes et al. (2020) argued that resilience is a dynamic rather than a linear process, and that it can evolve in response to shifts in people and their environmental subsystems. Therefore, there may also be individual differences in the relationship between resilience and creativity. De Caroli and Sagone (2014) found a significant relationship between creative personality traits and resilience in adolescents, and the more creative individuals perceived themselves to be, the more they tended to work on finding new solutions to their problems in a resilient way. Thus, the effect of resilience on creativity may vary due to the individuals’ own level of creativity and thus the differences in impact. As far as the relationship between academic self-efficacy and creativity is concerned, Haase et al. (2018) analyzed that most of the existing studies present a positive correlation between creative performance and creative self-efficacy, but the results of these studies indicate that the strength of the association varies and that there will be variability in the individual impact due to the influence of some moderating variables. So, we propose hypothesis H5, H6, H7:

**H5:**  *Effects of academic challenge stress on self-rated creativity are different.*

**H6:**  *Effects of resilience on self-rated creativity are different.*

**H7:**  *Effects of academic self-efficacy on self-rated creativity are different.*

This study is based on the interaction theory of creativity (Woodman et al. 1993) and social cognitive theory (Bandura 2002). According to the interaction theory of creativity, graduate students’ creativity is the result of the interaction between individual factors and situations, such as cognitive styles and abilities, knowledge base, motivation, social rewards, and organizational stressful environments. Academic challenge stress comes from the academic task, such as the research burden, academic demands, and time stress (Cavanaugh et al. 2000). The external positive pressure stimulates individual noncognitive factors in graduate students (resilience) and thus enhances creativity. Meanwhile, social cognitive theory emphasizes that self-efficacy is a key factor connecting the external environment and individual behavior. Because self-efficacy is the key driving force for individuals to set challenging goals for themselves and persevere in the face of difficulties, individuals with high self-efficacy are more likely to set challenging goals to change the status quo and generate novel and useful ideas. 

## 2. Materials and Methods

### 2.1. Participants

This study mainly collected data from postgraduates studying in universities in three provinces at eastern China including Jiangsu, Zhejiang, and Shanghai. Considering the stratification of the Chinese higher education system, a random sampling method based on the institutional level was used to conduct a questionnaire survey. We contacted the university’s information technology department and distributed the questionnaire to students after obtaining official approval. The link to the online survey was sent to graduate students via academic email. A total of 1210 valid questionnaires were collected after 1250 were distributed, with the effective response rate of the questionnaire being 96.8%. The demographic statistics of the participants included 42.9% (*n* = 519) men and 57.1% (*n* = 691) women in terms of gender. As for degree level, 61.7% (*n* = 747) of the respondents were master’s students and 38.3% (*n* = 463) were doctoral students. Graduate students in humanities (literature, history, and philosophy) accounted for 22.2% (*n* = 69), social sciences (economics, management, law, education, and society) accounted for 45.9% (*n* = 555), and science, agriculture, and medicine accounted for 21.9% (*n* = 386). The average age of the participants was 25.50 years (*SD* = 2.97).

We only conducted questionnaires that did not involve human physiology experiments and that guaranteed anonymity. When questionnaires were distributed, there was a consultation to fully inform all participants of the intention of the study and the use of the data. The research statement noted that (1) this survey is not about personal illnesses, habits, and other private issues but is only about personal aspects of mindfulness and self-rated creativity; (2) this survey is anonymous and no personal information will be published; (3) the raw data of this survey will not be used publicly; it will only be used by our research team. And only after seeking the consent of the graduate students by their ticking the “I voluntarily participate in being surveyed” option could they fill in the questionnaire further.

### 2.2. Materials

Participants completed Chinese versions of the academic challenge stress scale, the graduate student self-rated creativity scale, academic self-efficacy scale, and the resilience scale.

#### 2.2.1. Academic Challenge Stress

This study is based on the academic challenge stress scale developed by Cavanaugh et al. (2000), which has also been widely used in studies in China (Cai et al. 2022). Since the scale refers to challenge stress in general, this study limits challenge stress to the academic field. For example, we modified “I have great responsibility” to “I have great academic responsibility”. Five items were developed. Academic challenge stress is mainly related to taking on heavy academic responsibilities, undertaking challenging academic tasks, mastering multiple research methods, and completing high academic tasks and workload. Sample items are “I often have challenge academic responsibilities” and “I am required to complete a high level of academic tasks and workload within a specified time”. Participants were asked to rate these items on a 5-point scale, ranging from 1 (strongly disagree) to 5 (strongly agree). Cai et al. (2022) reported high internal consistency (α = 0.76) and average variance extracted (AVE = 0.569), which proved the reliability and construct validity of academic challenge stress. In this study, the Cronbach’s alpha coefficient of the academic challenge stress scale is 0.959, with a high internal consistency and Kaiser–Meyer–Olkin (KMO) = 0.901, Bartlett test *p* < 0.001, which suggests high inter-item correlations. In the CFA model, the relationship between each item and its respective latent variable was statistically significant, with all indicators loading exceeding 0.6. 

#### 2.2.2. Self-Rated Creativity

The self-rated creativity scale for graduate students uses the 6 items developed by Madjar et al. (2011), whose key indicators are creative thinking, solutions, and teamwork. Sample items are “I can formulate original and relevant research questions” and “I demonstrate originality in teamwork”. Participants are asked to rate these items on a 5-point scale, ranging from 1 (strongly disagree) to 5 (strongly agree). The scale has been translated into Chinese and applied to measure graduate students’ self-rated creativity (Tang and Ding 2014). Tang and Ding (2014) reported high internal consistency (α = 0.86). In this study, the Cronbach’s alpha coefficient of the scale is 0.950, with a high internal consistency, with Kaiser–Meyer–Olkin (KMO) = 0.904, Bartlett-test *p* < 0.001, which suggests high inter-item correlations. In the CFA model, the relationship between each item and its respective latent variable was statistically significant, with all indicators loading exceeding 0.6.

#### 2.2.3. Academic Self-Efficacy

The academic self-efficacy scale was measured using a scale developed by Joo et al. (2000) consisting of 9 items taken from the self-efficacy subscale of the motivation strategies for learning questionnaire and modified for graduate students. The scale has also been modified to fit Chinese schools (Zhu et al. 2011). Sample items are “I am sure I can do well in academic tasks” and “I enjoy the challenge of difficult academic tasks.” Participants answered questions on a 5-point scale from 1 (strongly disagree) to 5 (strongly agree) based on their expectations and beliefs about learning and research. This scale was validated by prior research (e.g., Bong 1997; Joo et al. 2000; Pintrich and De Groot 1990). Zhu et al. (2011) claimed that the scale had good internal consistency (α = 0.90). In this study, the scale had a Cronbach’s alpha value of 0.913, which indicated a high internal consistency, with Kaiser–Meyer–Olkin (KMO) = 0.911, Bartlett-test *p* < 0.001, which suggests high inter-item correlations. In the CFA model, the relationship between each item and its respective latent variable was statistically significant, with all indicators loading exceeding 0.6.

#### 2.2.4. Resilience

The resilience scale was measured using the resilience scale for youth and children developed by Mu and Hu (2016), consisting of 12 items. This study limited resilience to the academic field and therefore partially modified the questionnaire. We changed the item “My parents/caregivers know a lot about me” to “My scientific supervisor knows a lot about me”. In addition, in the sentence “I know where to go in the community to get help”, the word “community” is changed to “school”. Sample items are “I try to finish what I start” and “I am able to solve problems without harming myself or others.” Participants were asked to respond based on their own daily experiences, rating these items on a 5-point scale from 1 (strongly disagree) to 5 (strongly agree). Mu and Hu (2016) proved the scale’s consistency (α = 0.92), and they used this scale to test the assumption of psychometric invariance across various demographic cohorts. The questionnaire has also been used in some Chinese educational fields (Mu et al. 2017; Ma et al. 2021). In this study, the scale had a Cronbach’s alpha value of 0.925, with high internal consistency and Kaiser–Meyer–Olkin (KMO) = 0.914, Bartlett-test *p* < 0.001, which suggests good inter-item correlations. In the CFA model, the relationship between each item and its respective latent variable was statistically significant, with all indicators loading exceeding 0.6.

The item reliability and convergence validity were tested using CFA (Table 1). AMOS was used to estimate data and model fit. As for the CFA metrics, item reliability was tested using standardized factor loadings, which at a minimum need to be greater than 0.6 and significant (*p* < 0.05, Z > 1.96). Z-value refers to the standardized path coefficient divided by the standard error, representing the significance of each measurement topic. According to the statistical criteria, square multiple correlations (SMC) are required to be higher than 0.5. The average variance extracted (AVE) value reflects convergence validity, and it should be greater than 0.5 (Fornell and Larcker 1981; Hair et al. 1998). The results of the CFA are listed in Table 1. All of the indicators were better than the recommended standard values.

### 2.3. Data Analysis

SPSS 21 and AMOS 23 were used as analysis software for this study. Firstly, descriptive statistics analysis was conducted using SPSS to reflect the mean and standard deviation of each variable in this study. The Pearson correlation test was used to analyze the correlation between graduate students’ academic challenge stress, academic self-efficacy, self-rated creativity, and resilience. Secondly, a structural equation model was used to examine the latent variable path relationship between four variables. The bootstrap and Sobel’s test method were used to test the mediating effects. We used 5000 times of bootstrapping to estimate the coefficient values and standard errors and then used Sobel’s test to calculate the mediation effect values of each mediator variable. Since the AMOS software could not directly estimate the mediation effect values of each mediator variable in the case of the multiple mediators, we used the User-defined estimand in AMOS and edited the corresponding code to conduct the analysis. After obtaining the total mediator effect value and each mediator effect value, the mediator effect values of resilience and academic self-efficacy and the chained mediator effect values of the two were divided by the total mediator effect value to obtain the percentage of each mediator effect degree.

Regarding conducting structural equation modeling, CFI, TLI, SRMR, and RMSEA are commonly used as the fit indices that must be reported in general research. The maximum likelihood (ML) method was used as an estimation method. We tested the premise of ML estimation, that is, the sample conforms to the normal distribution. We used AMOS 23 for testing, the absolute values of skewness and kurtosis were <3.00 (Kline 2011). In addition, the Mardia’s coefficient of multivariate kurtosis (549.110) was <*p* (*p* + 2) (*p* = the number of observed variables = 32) = 1088 (Nimon 2012; Mardia 1985). These findings confirmed the univariate and multivariate normal distribution of the data. The following criteria are used to determine whether the data conform well to the hypothetical model. Sharma et al. (2005) concluded that when the sample size is greater than 200 and the factor loadings are higher than 0.5, the performance of the TLI test model fit is the best among all the groups of test metrics, with a suggested threshold criterion of 0.9. A rule of thumb is that the SRMR should be less than 0.05 for a good fit (Hu and Bentler 1995), whereas values smaller than 0.10 may be interpreted as acceptable (Schermelleh-Engel et al. 2003). According to Browne and Cudeck (1992), RMSEA values ≤ 0.05 can be considered as a good fit, values between 0.05 and 0.08 as an adequate fit, and values between 0.08 and 0.10 as a mediocre fit, whereas values > 0.10 are not acceptable. The CFI is relatively more biased toward determining model fitness independent of sample size. The qualification standard for CFI is 0.9 (McDonald and Ho 2002).

## 3. Results

### 3.1. Descriptive Statistics and Correlation Tests

Table 2 presents the descriptive results and correlations of the investigated variables. Academic challenge stress was significantly and positively correlated with graduate student self-rated creativity. It also showed significant positive correlations with both academic efficacy and resilience. Academic efficacy and resilience also showed significant positive correlations with graduate student self-rated creativity.

### 3.2. Testing Latent Variable Path Model and Mediation Effects

**Figure 2:**
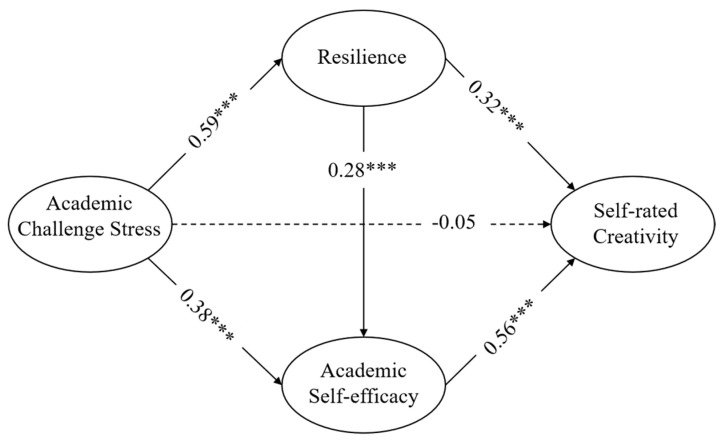
The path coefficients of model. *** *p* < 0.001.

In the structural model, as shown in Figure 2, the result of the model fitting index indicated that the data conformed to the hypothetical model (CFI = 0.94, TLI = 0.93, RMSEA = 0.07 [90% CI = 0.072, 0.077], SRMR = 0.05). The model latent variable path coefficient results suggested that academic challenge stress was positively related to resilience (β = 0.59, *p* < 0.001) and academic self-efficacy (β = 0.38, *p* < 0.001). Meanwhile, resilience (β = 0.32, *p* < 0.001) and academic self-efficacy (β = 0.56, *p* < 0.001) were also significantly and positively correlated with self-rated creativity, and resilience was positively related to creative self-efficacy (β = 0.28, *p* < 0.001). But in the impact path, the impact of academic challenge stress on graduate self-rated creativity was no longer significant, which suggested that resilience and academic self-efficacy might fully mediate the relationship between challenge academic stress with self-rated creativity in this model.

The bias-corrected nonparametric percentile Bootstrap method was applied to test for mediating effects (Wen et al. 2010). Specifically, 95% confidence intervals for mediating effects were estimated (see Table 3). The results showed that the total effect of academic challenge stress and creativity was significant and positive, and hypothesis 1 was supported. The mediating effects for both resilience (β = 0.188, Z > 1.96, 95% CI = [0.146, 0.228]) and academic efficacy (β = 0.216, Z > 1.96, 95% CI = [0.172, 0.273]) reached statistical significance. Resilience and academic efficacy had mediating effect sizes of 37.7% and 43.3%, respectively. The chained mediating effect size was 19.0%. The mediation effect of resilience was slightly lower than that of academic efficacy, but the difference was not significant when comparing the coefficients. The chain mediating effect of resilience and academic efficacy also held (β = 0.095, Z > 1.96, 95% CI = [0.066, 0.131]) with a mediating effect of 19.0%. Meanwhile, we calculated the direct effect of academic challenge stress on graduate student self-rated creativity and found that the direct effect was no longer significant (β = −0.050, Z > 1.96, 95% CI = [−0.135, 0.009]). Hypotheses 2, 3 and 4 were supported.

### 3.3. Heterogeneity Test: Based on Quantile Regression Model of Self-Rated Creativity

The structural equation model was able to reveal the effects of academic challenge stress, academic efficacy, and resilience on graduate student self-rated creativity only at the mean level, but it could not tell researchers whether the effects of the three factors on graduate student self-rated creativity were characterized by individual heterogeneity. To capture this heterogeneity characteristic, we chose quartiles 10 to 90 to indicate the variability of the effect of student self-rated creativity at low to high quartiles respectively and used conditional quantile regression to obtain regression results for the heterogeneity analysis by bootstrap sampling 1000 times.

**Figure 3:**
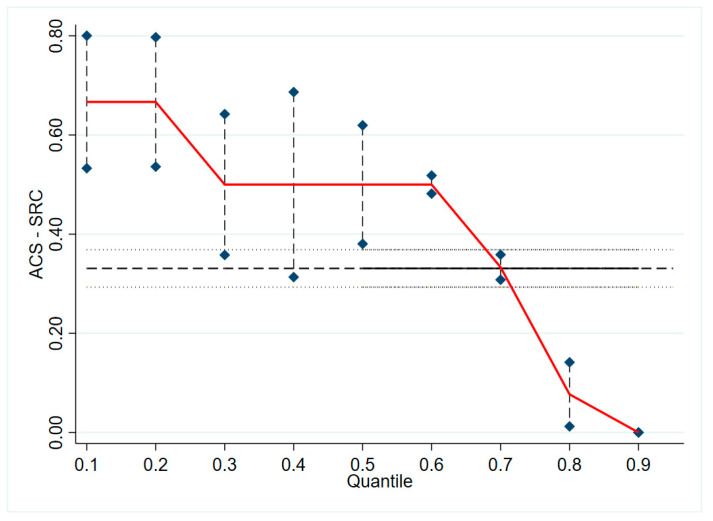
Heterogeneity of the effects of academic challenge stress on students with different self-rated creativity.

**Figure 4:**
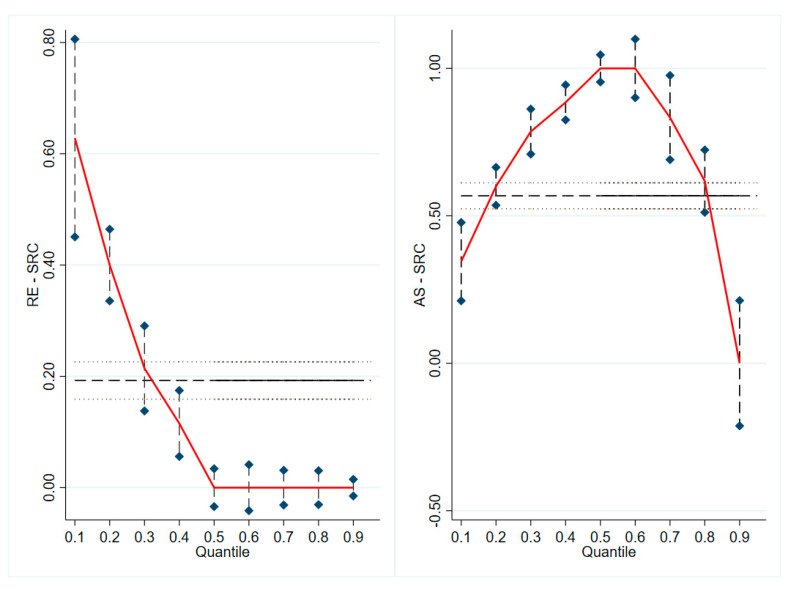
Heterogeneity of the effects of resilience and academic efficacy on students with different self-rated creativity.

The results are shown in Figure 3 and Figure 4. Figure 3 shows the differences in the effects of academic challenge stress on different graduate students. The diamond-shaped dashed line in the figure indicates 95% confidence intervals of the parameter estimates. The red line indicates parameter estimates at the different regression quantiles. The black horizontal line indicates parameter estimates for the ordinary linear regression with the same predictors. The two black dotted lines above and below the black solid line indicate upper and lower confidence intervals for parameter estimates for the OLS.

It can be found that the effects of academic challenge stress on graduate students’ self-rated creativity have diminishing marginal benefits, i.e., for graduate students with relatively low self-rated creativity, academic challenge stress can have a greater effect on self-rated creativity enhancement (β = 0.66, *p* < 0.001), while for graduate students with relatively high self-rated creativity, the effect of academic challenge stress on self-rated creativity enhancement was not significant (β < 0.10, *p* > 0.05). Hypothesis 5 was supported.

Figure 4 shows the differences in the effects of resilience and academic efficacy on different graduate students. The effects of resilience on graduate student self-rated creativity also have diminishing marginal benefits. For graduate students with relatively low levels of self-rated creativity, an increase in the level of resilience was effective in enhancing graduate student self-rated creativity (β = 0.62, *p* < 0.001). But for graduate students with relatively high levels of self-rated creativity, an increase in the level of resilience did not lead to a sustained increase in self-rated creativity (β = 0.01, *p* > 0.05). The effect of academic self-efficacy on self-rated creativity of graduate students showed an inverted U-shaped relationship, i.e., the effect of academic self-efficacy on self-rated creativity was low for graduate students with higher and lower levels of self-rated creativity, but the effect of academic self-efficacy on self-rated creativity was greatest for graduate students with moderate levels of self-rated creativity (β = 0.99, *p* < 0.001). Hypotheses 6 and Hypotheses 7 were supported.

## 4. Discussion

### 4.1. Academic Challenge Stress and Graduate Student Self-Rated Creativity

The results of this study suggest that academic challenge stress helps to enhance graduate student self-rated creativity. This finding is similar to previous studies, but previous research on this topic focused on the corporate employee level (Ren and Zhang 2015; Sun et al. 2019; Cai et al. 2022; Moin et al. 2022), suggesting that the findings have applicability to university graduate students as well. Graduate students who perceive themselves as capable of handling daily academic stress stimuli see academic stress as an opportunity for growth, and academic challenge stress is a “rewarding” stress (Cavanaugh et al. 2000). Drawing on expectancy theory, LePine et al. (2005) argue that challenge stressors generate positive motivation and that individuals’ efforts in the face of such stressors can ultimately lead to valuable outcomes. Challenge stress has a more positive effect on motivation, work attitude, and performance, and it triggers personal desire and self-confidence to adopt creative problem-solving strategies (Wallace et al. 2009; He et al. 2019). In the process, self-rated creativity is effectively enhanced. In conclusion, managing academic stress is not simply eliminating the stressors, but maintaining the appropriate stress so that stress and motivation can coexist. 

### 4.2. The Mediating Effects of Academic Self-Efficacy and Resilience

The study found that academic challenge stress affects self-rated creativity through a complete chain of mediating effects of graduate student resilience and academic efficacy. Previous related studies have mostly focused on the exploration of moderating variables (Byron et al. 2018; Liu and Li 2018) and few studies revealed the mediating mechanisms by which stress affects self-rated creativity. This study presents a new theoretical explanation of the underlying mechanism of the influence of academic self-efficacy and resilience as the core variables to explain the relationship between academic challenge stress and self-rated creativity.

Whereas academic self-efficacy as a mediating role also has a plausible explanation, challenge stressors have been shown to lead to positive affective and attitudinal responses (LePine et al. 2016; Prem et al. 2017). This finding is also in line with Bandura’s theory of efficacy regarding the interplay between individual cognition, context, and behavior. The importance of individual initiative in context and the role of individual self-efficacy should be emphasized when discussing the relationship between stress and self-rated creativity (Liu et al. 2016; He et al. 2019).

Resilience, an important component of the psychological capital of graduate students in higher education, enables individuals to persevere, recover, and grow quickly in the face of adversity or challenge stress. In fact, a study by García-Rivera et al. (2022) found that resilience was indeed effective in promoting mental health in individuals. According to resource conservation theory (Hobfoll et al. 2018), resilience can be increased or decreased in different external environments (Hobfoll et al. 2015). Living in a resource-rich environment will accumulate resource gains and promote resilience. When graduate students face academic challenge stress, overcoming the stress generates positive emotional resources that promote resilience. According to emotional motivation theory (Gable and Harmon-Jones 2010), people with more resilience tend to maintain a positive steady state and come up with creative problem-solving solutions (Avey et al. 2011; Tong et al. 2022).

### 4.3. Heterogeneity of Self-Rated Creativity Influence Mechanisms

It was found that the effects of academic challenge stress, resilience, and academic self-efficacy on promoting graduate student self-rated creativity were not always constant but had differential effects on graduate students of different self-rated creativity levels. Specifically, academic challenge stress produces greater benefits for less creative graduate students. They lack a certain level of academic research autonomy, needing to exert external stress to enhance students’ academic efficacy and promote their achievement motivation (Bockorny et al. 2023). Similarly, resilience has a greater benefit for graduate students with lower self-rated creativity and does not have a positive effect on those with higher self-rated creativity. This is because graduate students with higher self-rated creativity, who have already taken on certain academic challenge stress, have higher resilience so that raising their resilience again could not effectively promote their self-rated creativity again. In contrast, there is an inverted U-shaped relationship between academic self-efficacy and self-rated creativity, which indicates that academic efficacy is most effective for graduate students who have a moderate level of self-rated creativity.

### 4.4. Educational Practice Implications

This study has important implications and references for educational practices. First, the positive relationship between challenge stress and creativity has been demonstrated in the field of business management (Sun et al. 2019; Cai et al. 2022) but has yet to be expanded in the field of graduate education. In the context of massive expansion of graduate students globally (Sarrico 2022), how universities can enhance graduate students’ creativity for innovation output is of significance for universities worldwide. This study confirms that academic challenge stress is not a negative stress; on the contrary, appropriate academic challenge stress can promote graduate student creativity. Second, based on the interaction theory and social cognitive theory of creativity, this study applies the theory to the field of graduate education and supports the mechanism of resilience and academic self-efficacy in the influence of academic challenge stress on creativity. This finding can help university leaders and instructors to understand how university organizations can enhance graduate student creativity by setting appropriate academic challenge stress.

The current study also offers major practical implications for supervisors in higher education. In order to enhance the creativity of graduate students, graduate student supervisors should not give no or heavy research stress but should reasonably set academic challenge stress and academic challenge goals. At the same time, resilience and academic self-efficacy are malleable personal traits that play an important mediating role in the process of academic challenge stress affecting creativity. As innovative activities are full of unknowns and uncertainties, resilience and academic self-efficacy are important drivers for graduate students to break the boundaries of their thinking and behaviors in accomplishing their tasks. So, advisors can guide graduate students to participate in learning activities and create a positive organizational climate for innovation and focus on the construction of the research environment. As for institutions, enriching academic communication resources and setting goal rewards for graduate students are necessary so as to maximize the intrinsic value of challenge stress. In addition, attention should be paid to the differences among individuals. Different academic challenge goals and incentives for graduate students with different creativity levels are needed.

### 4.5. Limitations and Future Directions

This study has limitations in the following aspects. First, this study is based on questionnaire data at one point in time and lacks research on causal relationships over long time periods. The relationship effects between variables in the model may change when spanning a longer period of time, e.g., graduate students may have different impact outcomes on self-rated creativity in the process of facing academic challenge stress over a long period of time. A longitudinal research design should be further used to determine the relationship between variables in future studies. Second, there are limitations to our methodology. We selected two very representative mediating variables, resilience and academic self-efficacy, based on literature and theory. However, due to data and technical limitations, we have not considered other possible mediating variables in the questionnaire design, and therefore, we could not eliminate the most probable threats to internal validity (e.g., confounder variables in mediation models and competitive mediation models). Hence, future studies will incorporate more mediating variables in order to compare the effects exerted by different mediating variables and to verify the optimal mediating mechanism. Finally, although the study clarified the relationship between the effects of academic challenge stress on graduate students’ self-rated creativity and the mediating effects of resilience and academic self-efficacy, the underlying mechanisms of their effects can be theoretically explored in more depth and richer relationships of variables can be sought.

## 5. Conclusions

This study found that academic challenge stress was significantly positively related to graduate student self-rated creativity. The effect of academic challenge stress on graduate students’ self-rated creativity was influenced through the fully mediated effect of resilience and academic efficacy, and the mediated effect of academic efficacy was slightly greater than that of resilience. In addition, the quantile regression results well reflect that there are differences in the effects of academic challenge stress, resilience, and academic self-efficacy on promoting graduate students’ self-rated creativity. Academic challenge stress and resilience both have better effects on graduate students with relatively low levels of self-rated creativity, while academic self-efficacy is most effective on graduate students with moderate levels of self-rated creativity. 

## Figures and Tables

**Table 1:** Confirmatory factor analysis.

Measure	Measurement Items Z-Value	Standardized Factor Loadings	Square Multiple Correlations	Average Variance Extracted
Academic Challenge Stress	45.555–58.779	0.854–0.934	0.729–0.872	0.816
Self-rated creativity	38.044–88.267	0.763–0.976	0.582–0.953	0.837
Academic self-efficacy	33.103–58.750	0.739–0.959	0.546–0.920	0.821
Resilience	32.079–43.558	0.783–0.949	0.613–0.901	0.747

**Table 2:** Means, standard deviations, and correlations among variables in the research.

Variable	1	2	3	4
1. Academic Challenge Stress	-			
2. Self-rated creativity	0.443 **	-		
3. Academic Self-efficacy	0.555 **	0.701 **	-	
4. Resilience	0.585 **	0.543 **	0.503 **	-
Mean	3.846	4.276	4.258	3.674
Standard Deviation	0.842	0.629	0.634	0.833

Note: ** *p* < 0.01.

**Table 3:** Mediating results in the SEM.

Mediation Effect Test	Estimation	BootStrap SE	Z	Bootstrapping 95% CI		*p*	Mediation Effect Proportion
				Lower	Upper		
Total effect	0.449	0.029	15.483	0.393	0.504	-	-
Direct effect	−0.050	0.034	−1.471	−0.135	0.009	-	-
Total indirect effect	0.499	0.029	17.207	0.441	0.555	0.007	-
Indirect effect (ACS→RE→SRC)	0.188	0.022	8.545	0.146	0.228	0.012	37.7%
Indirect effect (ACS→AS→SRC)	0.216	0.026	8.308	0.172	0.273	0.004	43.3%
Indirect effect (ACS→RE→AS→SRC)	0.095	0.016	5.938	0.066	0.131	0.008	19.0%
Compare with RE-AS	−0.028	0.035	−0.800	−0.103	0.036	0.473	-

Note: SE-Standard Error, ACS means academic challenge stress, RE means resilience, AS means academic self-efficacy, SRC means self-rated creativity. Mediation effect proportion refers to the proportion of the mediating variable’s mediation effect to the total mediation effect (individual mediation coefficients divided by total mediation coefficients).

## Data Availability

Data are protected due to student privacy; for access to the data, please contact the corresponding author.
